# Supplementary material for: Pianno: a probabilistic framework automating semantic annotation for spatial transcriptomics
Source: Nat Commun. 2024 Apr 2;15:2848. doi: 10.1038/s41467-024-47152-4 (PMC11271244; doi:10.1038/s41467-024-47152-4)
Supplement: Supplementary file 1 — Supplementary Information [file 41467_2024_47152_MOESM1_ESM.pdf]

Supplementary Materials for

Pianno: a probabilistic framework automating semantic annotation for spatial transcriptomics

Yuqiu Zhou[1], Wei He[1], Weizhen Hou[1], Ying Zhu[1*]

[1*]State Key Laboratory of Medical Neurobiology, MOE Frontiers Center for Brain Science, Institutes of Brain Science and Department of Neurosurgery, Huashan Hospital, Fudan University, Shanghai, 200032, China.

*Corresponding author(s). E-mail(s): ying_zhu@fudan.edu.cn;

Supplementary Note

Transformation of spatial coordinates to image coordinates

There are usually two forms of spatial coordinates, one is an array-like row index, and the other is a pixel-level physical coordinate. For the former Mask is relatively simple and intuitive to construct. However, high-resolution spatial transcriptome techniques usually only have finer pixel-level physical coordinates, such as SlidseqV2, and therefore require the user to specify a scale factor (which defaults to 1 for array-like coordinates)The coordinate conversion is performed according to the following equation.

$$\begin{cases} i = \mathrm{int}(\frac{x_s - \min_{\forall s \in \mathbb{S}}\{x_s\}}{\mathrm{scalefactor}}) \\ j = \mathrm{int}(\frac{y_s - \min_{\forall s \in \mathbb{S}}\{y_s\}}{\mathrm{scalefactor}}) \end{cases} \quad (1)$$

where int(·) denotes the rounding function in Python, and the formula above is equivalent to binning for spots.

Supplementary Figures

Fig. S1 Comparison of spatial domains by clustering assignments via SpaGCN, SEDR, BayesSpace, DeepST, STAGATE, Pianno and manual annotation in all 12 samples of the human dlPFC dataset.
Fig. S2 Unsupervised clustering label mapping via Kuhn-Munkres Algorithm.
Fig. S3 Pianno's performance in structure and cell-type probability inference.
Fig. S4 Pianno annotation for MOB.
Fig. S5 Multimodal data exploratory analysis with Pianno.
Fig. S6 Sensitivity analysis.

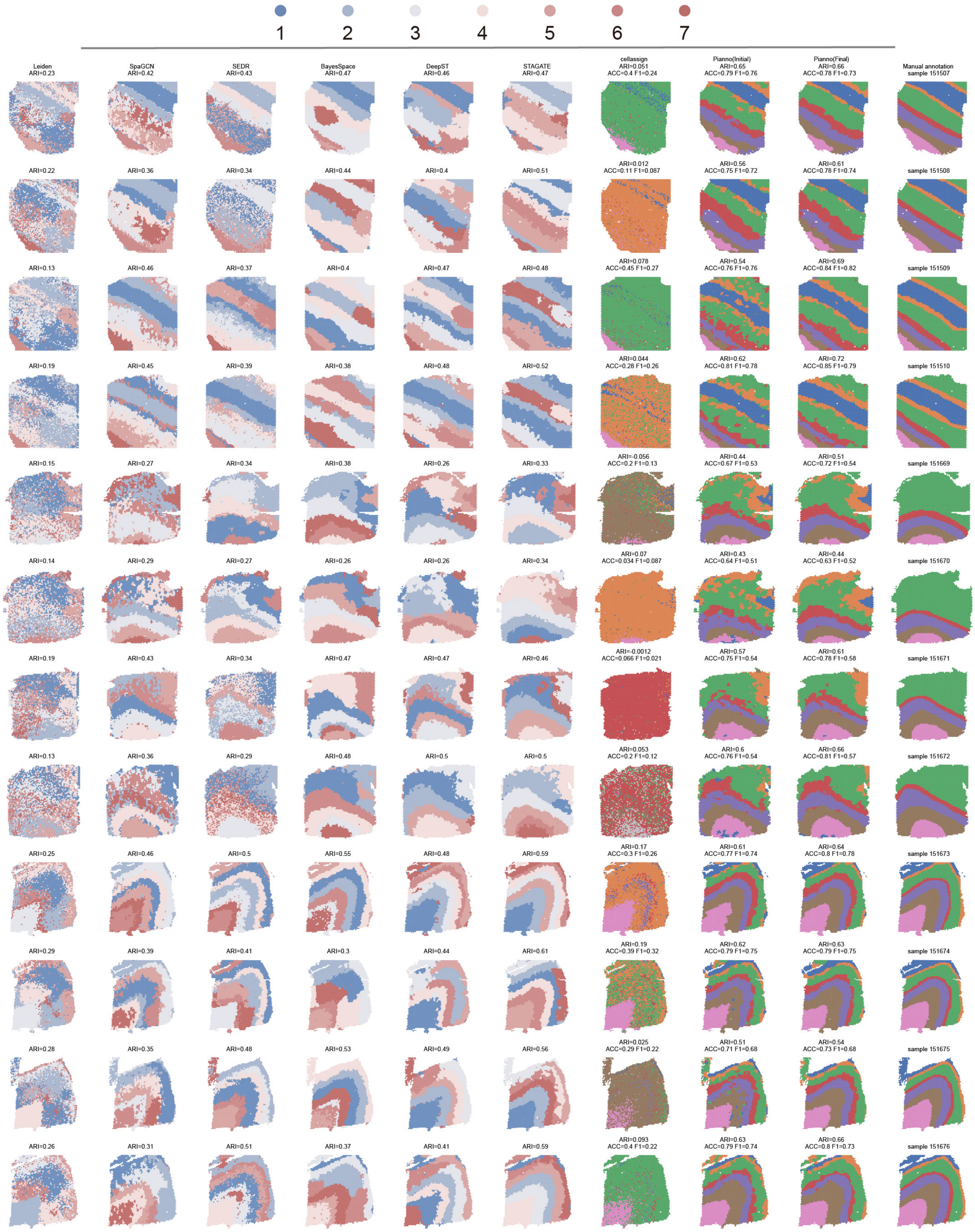

Fig. S1 Comparison of spatial domains by clustering assignments via SpaGCN, SEDR, BayesSpace, DeepST, STAGATE, Pianno and manual annotation in all 12 samples of the human dlPFC dataset.

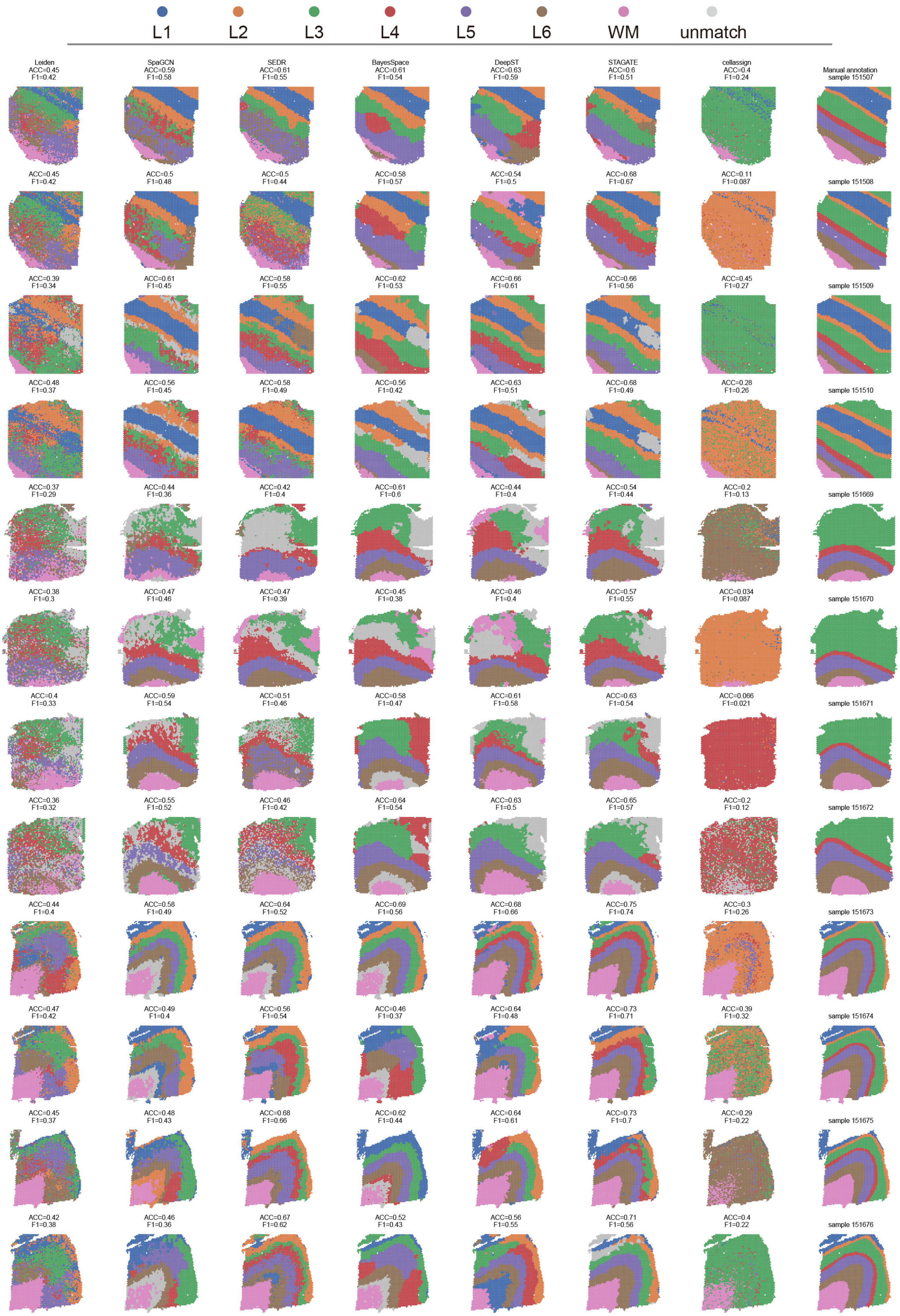

Fig. S2 Unsupervised clustering label mapping via Kuhn-Munkres Algorithm. Source data are provided as a Source Data file.

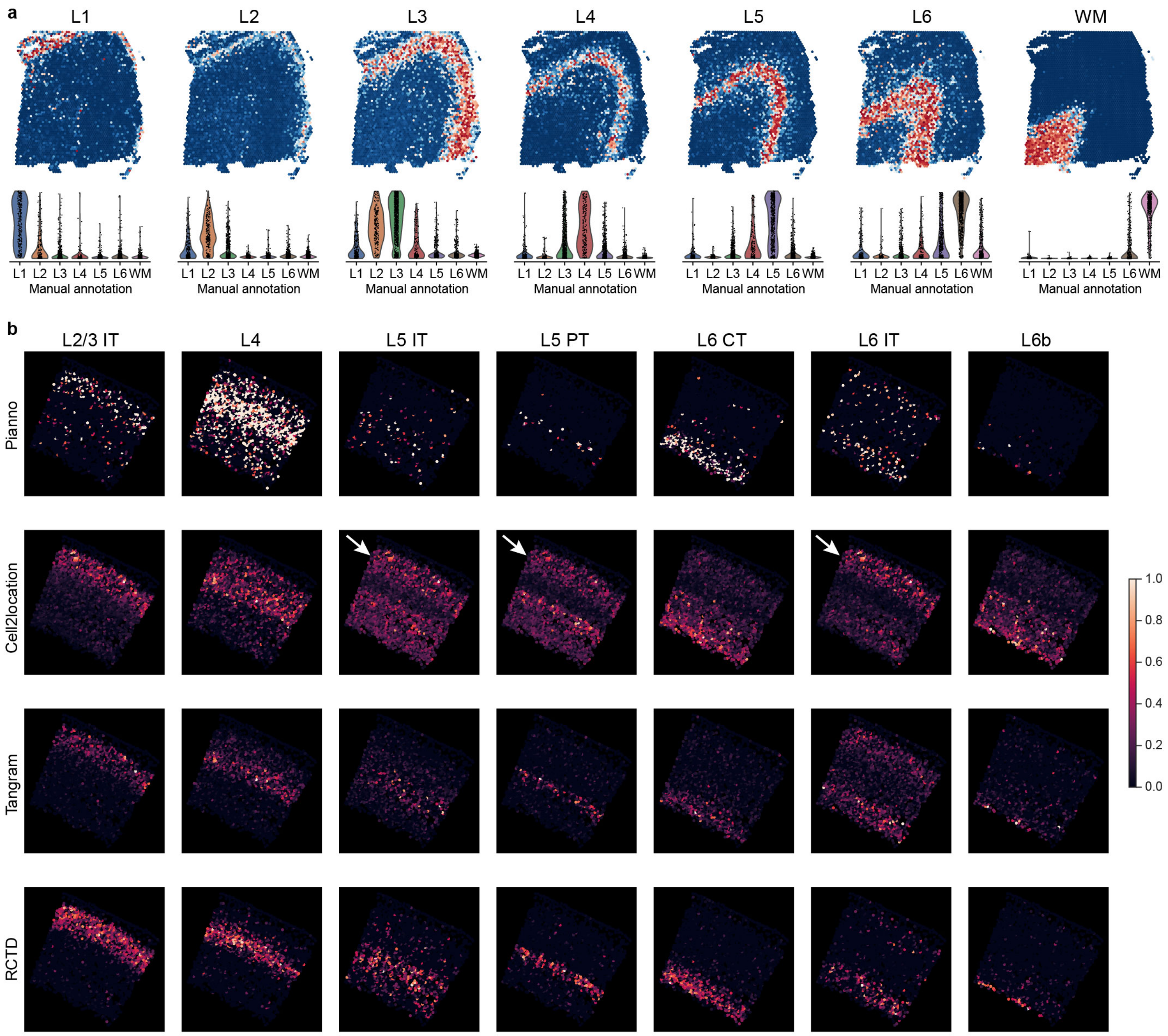

Fig. S3 Pianno's performance in structure and cell-type probability inference. a, Posterior probability distribution in dlPFC sample 151673 inferred by Pianno. Top: posterior probability heatmap of different cortical structures. Bottom: violin plots of Pianno's inferred structure probability distributions in manually annotated cortical structures. **b**, The distribution probability of selected cell types inferred by Pianno, Cell2location, Tangram, and RCTD. Source data are provided as a Source Data file.

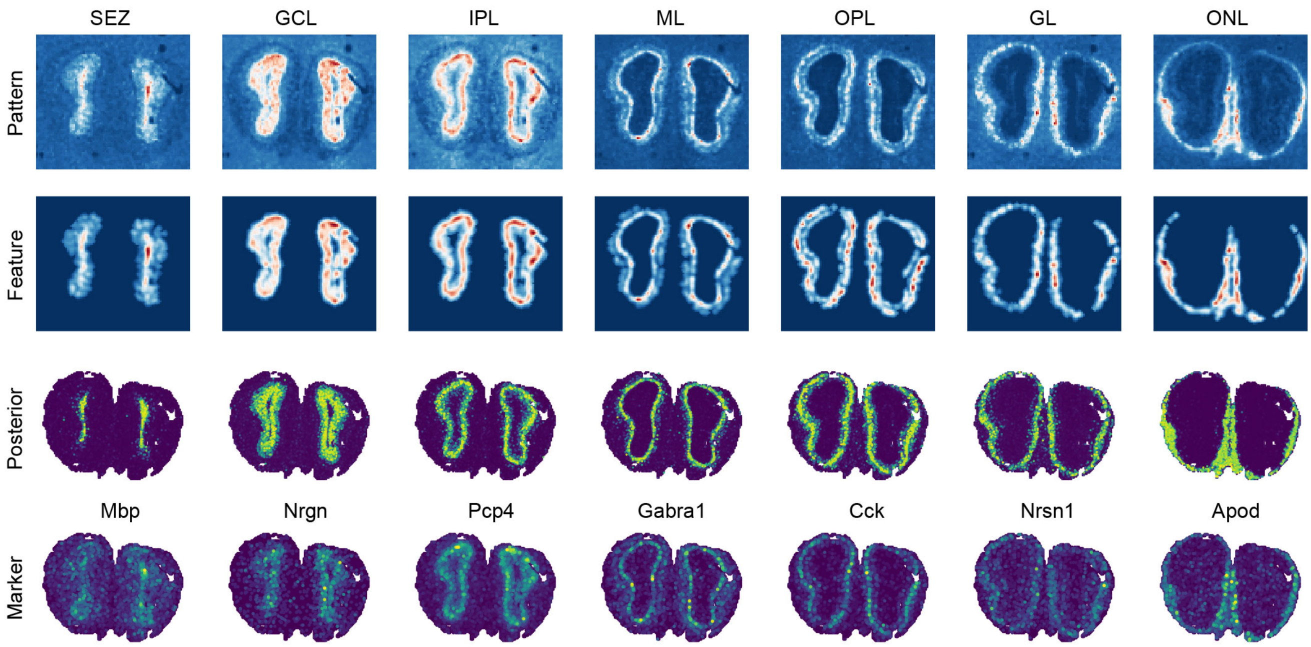

Fig. S4 **Pianno annotation for MOB.**

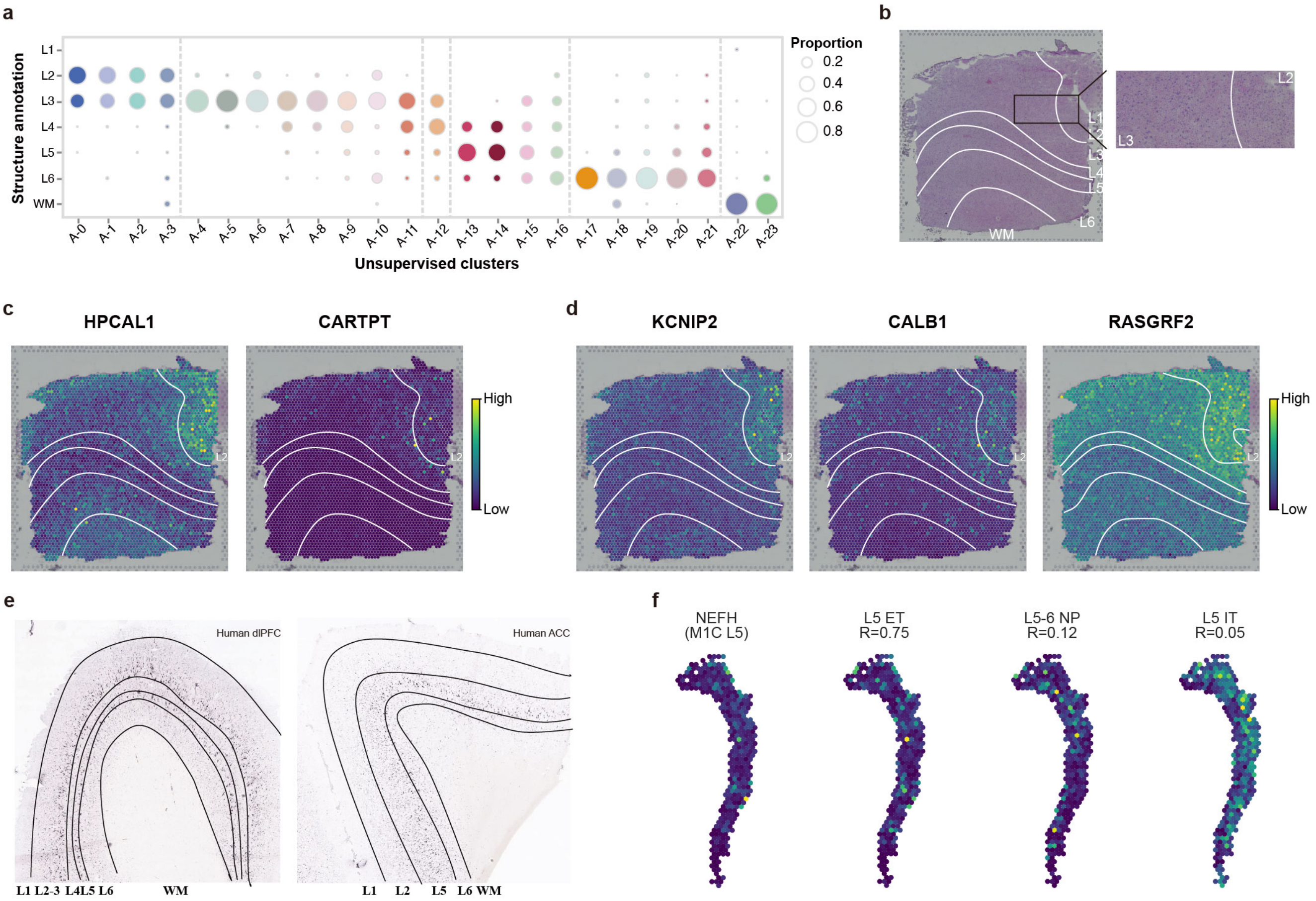

Fig. S5 Multimodal data exploratory analysis with Pianno. **a**, Proportion of unsupervised clusters within each structure. **b**, H&E stained image of human dlPFC sample 151671. The white lines delineated the boundaries between layers annotate by Pianno (right). Histological difference between L2 and L3 is highlighted and displayed (left). **c**, Visualization of L2 markers used during Pianno annotation of sample 151671. **d**, Visualization of known L2 markers not used during Pianno annotation of sample 151671. Those markers were found in the DEGs of L2 from sample 151671. **e**, Validation of laminar distribution of $NEFH^+$ in human dlPFC (left) and ACC (right). The lines delineated the boundaries between layers. The ISH images were downloaded from Allen Brain Atlas. **f**, Pearson correlation coefficients (R) between the *NEFH* expression pattern and the probability distribution of each L5 excitatory neuron subtypes in M1C L5.

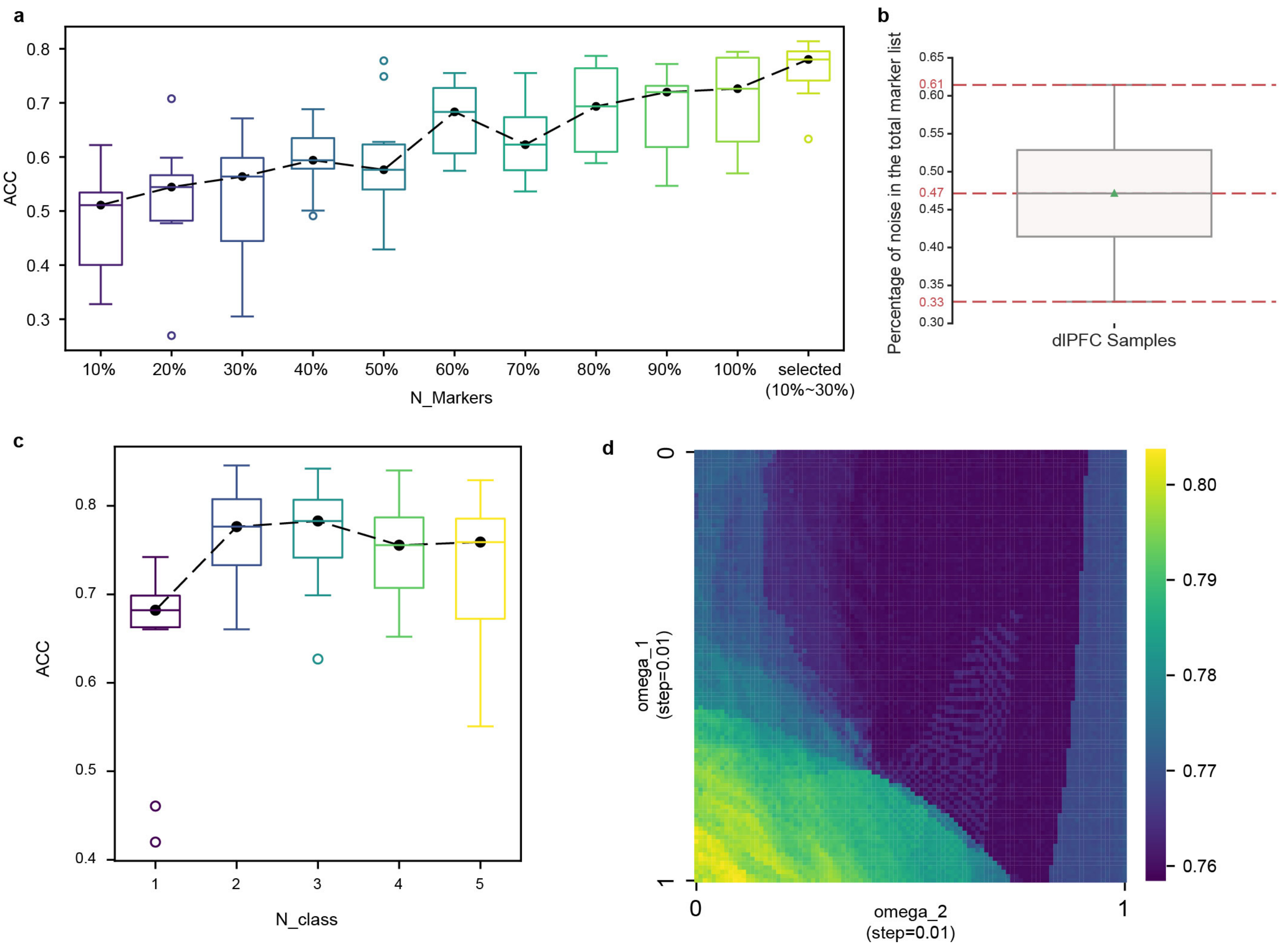

Fig. S6 Sensitivity analysis. **a**, Accuracy of Pianno prediction on dlPFC samples (n=12), using marker genes randomly selected from the total marker gene list (n=10). The box bounds the interquartile range (IQR) divided by the median, with the whiskers extending to a maximum of 1.5 × IQR from the box, and values beyond the whiskers are considered outliers, marked with hollow circles. **b**, For each dlPFC sample, the percentage of noise in the total marker list. **c**, Trend of accuracy of dlPFC samples (n=12) when the number of thresholds of the Multi-Otsu thresholding algorithm (N_class) increases from 1 to 5. **d**, Trend of the accuracy of Pianno annotation on dlPFC sample 151673 when the hyperparameters ω_1 and ω_2 in the High-Order MRF Prior Model increased from 0 to 1 with a step size of 0.01. Source data are provided as a Source Data file.
