# Supplementary material for: Pianno: a probabilistic framework automating semantic annotation for spatial transcriptomics
Source: Nat Commun. 2024 Apr 2;15:2848. doi: 10.1038/s41467-024-47152-4 (PMC11271244; doi:10.1038/s41467-024-47152-4)
Supplement: Supplementary file 3 — Description of Additional Supplementary Files [file 41467_2024_47152_MOESM3_ESM.pdf]

Description of Additional Supplementary Files:

Supplementary Data 1: presents the evaluation scores for all the methods used on the dlPFC dataset.

Supplementary Data 2: provides a list of the top 100 differentially expressed genes within clusters from the human dlPFC sample 151671.

Supplementary Data 3: provides a list of Gene Ontology (GO) terms enriched in cluster A-11 within the dlPFC sample 151671.

Supplementary Data 4: provides a list of the top 100 differentially expressed genes within clusters from the human M1C dataset.
