# Supplementary material for: Pianno: a probabilistic framework automating semantic annotation for spatial transcriptomics
Source: Nat Commun. 2024 Apr 2;15:2848. doi: 10.1038/s41467-024-47152-4 (PMC11271244; doi:10.1038/s41467-024-47152-4)
Supplement: Supplementary file 5 — Reporting Summary [file 41467_2024_47152_MOESM5_ESM.pdf]

nature portfolio

Corresponding author(s): Ying Zhu

Last updated by author(s): Mar 3, 2024

Reporting Summary

Nature Portfolio wishes to improve the reproducibility of the work that we publish. This form provides structure for consistency and transparency in reporting. For further information on Nature Portfolio policies, see our Editorial Policies and the Editorial Policy Checklist.

Statistics

For all statistical analyses, confirm that the following items are present in the figure legend, table legend, main text, or Methods section.

n/a	Confirmed	
☐	☒	The exact sample size (n) for each experimental group/condition, given as a discrete number and unit of measurement
☐	☒	A statement on whether measurements were taken from distinct samples or whether the same sample was measured repeatedly
☐	☒	The statistical test(s) used AND whether they are one- or two-sided<br>*Only common tests should be described solely by name; describe more complex techniques in the Methods section.*
☒	☐	A description of all covariates tested
☐	☒	A description of any assumptions or corrections, such as tests of normality and adjustment for multiple comparisons
☐	☒	A full description of the statistical parameters including central tendency (e.g. means) or other basic estimates (e.g. regression coefficient) AND variation (e.g. standard deviation) or associated estimates of uncertainty (e.g. confidence intervals)
☐	☒	For null hypothesis testing, the test statistic (e.g. F, t, r) with confidence intervals, effect sizes, degrees of freedom and P value noted<br>*Give P values as exact values whenever suitable.*
☐	☒	For Bayesian analysis, information on the choice of priors and Markov chain Monte Carlo settings
☒	☐	For hierarchical and complex designs, identification of the appropriate level for tests and full reporting of outcomes
☐	☒	Estimates of effect sizes (e.g. Cohen's d, Pearson's r), indicating how they were calculated

Our web collection on statistics for biologists contains articles on many of the points above.

Software and code

Policy information about availability of computer code

Data collection	No software was used.

Data analysis	R package:<br>scran(v1.28.2)<br>saver(v1.1.2)<br>BayesSpace(v1.5.1, https://github.com/edward130603/BayesSpace)<br>Python packages:<br>Pianno(v2.0.0 , https://github.com/yuqiuzhou/Pianno)<br>DeepST(https://github.com/JiangBioLab/DeepST)<br>STAGATE(v1.0.1, https://github.com/zhanglabtools/STAGATE)<br>SpaGCN(v1.2.7, https://github.com/jianhuupenn/SpaGCN)<br>SEDR(https://github.com/JinmiaoChenLab/SEDR)<br>scanpy(v1.9.1)<br>squidpy(v1.2.2)<br>scikit-learn(v1.0.2)<br>scikit-image(v0.19.2)<br>opencv-python(v4.5.5.64)<br>tensorflow-gpu(v2.6.0)<br>tensorflow-probability(v0.14.0)

For manuscripts utilizing custom algorithms or software that are central to the research but not yet described in published literature, software must be made available to editors and reviewers. We strongly encourage code deposition in a community repository (e.g. GitHub). See the Nature Portfolio guidelines for submitting code & software for further information.

Data

Policy information about availability of data

All manuscripts must include a data availability statement. This statement should provide the following information, where applicable:

- Accession codes, unique identifiers, or web links for publicly available datasets
- A description of any restrictions on data availability
- For clinical datasets or third party data, please ensure that the statement adheres to our policy

Spatial transcriptome data from the adult human primary motor cortex (M1C) and anterior cingulate cortex (ACC) generated for this study has been deposited in the Genome Sequence Archive (GSA) under accession HRA004425, and the processed datasets are available at https://github.com/yuqiuzhou/Pianno. All public datasets utilized in this study are accessible in their raw form from the respective original authors. Specifically, the human dlPFC dataset is available within the spatialLIBD (http://spatial.libd.org/spatialLIBD). The processed Stereo-seq datasets from adult mouse coronal hemibrain and olfactory bulb are available at the Spatial Transcript Omics DataBase (STOmics DB) (https://db.cngb.org/stomics). The pre-processed SlideseqV2 dataset from mouse hippocampus is accessible within the Squidpy package (https://github.com/scverse/squidpy). The ST datasets of human pancreatic ductal adenocarcinoma are available at the Gene Expression Omnibus under accession number GSE111672. The Visium datasets of human breast cancer are collected from the 10x Genomics website (https://support.10xgenomics.com/spatial-gene-expression/datasets). The scRNA-seq dataset from mouse primary visual cortex is available at the NCBI Gene Expression Omnibus (GEO) under accession GSE115746. The snRNA-seq dataset from multiple human cortical areas is available at Allen Brain Map (https://portal.brain-map.org/). The DAPI staining image of mouse olfactory bulb is accessible on https://github.com/JinmiaoChenLab/SEDR analyses.

Research involving human participants, their data, or biological material

Policy information about studies with human participants or human data. See also policy information about sex, gender (identity/presentation), and sexual orientation and race, ethnicity and racism.

Reporting on sex and gender	Sex and gender were not taken into consideration in this study.
Reporting on race, ethnicity, or other socially relevant groupings	No categorization on race, ethnicity, or other socially relevant groupings was defined or conducted in this study. The research utilized biological samples from a single human participant, and this individual's demographics were not pertinent to the specific questions being addressed in this study.
Population characteristics	A single healthy neocortical area from a 77-year-old female was utilized to generate spatial transcriptome data.
Recruitment	Postmortem specimens used to generate spatial transcriptome sequencing data were obtained from adult donors with clinically unremarkable backgrounds. The time elapsed since death (postmortem interval, PMI) was less than 24 hours, and all donor information was de-identified.
Ethics oversight	Ethical approval for this study was obtained from the School of Basic Medical Sciences, Fudan University (Approval No. 2020-C006).

Note that full information on the approval of the study protocol must also be provided in the manuscript.

Field-specific reporting

Please select the one below that is the best fit for your research. If you are not sure, read the appropriate sections before making your selection.

[x] Life sciences [ ] Behavioural & social sciences [ ] Ecological, evolutionary & environmental sciences

For a reference copy of the document with all sections, see nature.com/documents/nr-reporting-summary-flat.pdf

Life sciences study design

All studies must disclose on these points even when the disclosure is negative.

Sample size	No statistical method was used to predetermine sample size. The number of samples were chosen for this exploratory study based on the availability of materials at study time. The spatial transcriptome data generated in this study was isolated from a single donor as a validation.
Data exclusions	We did not include data that were clearly outliers in spatial transcriptome in the analysis. Low-quality genes detected in less than 1% of spots, as well as spots without gene counts detected, are sieved out.
Replication	All experimental steps are detailed in the manuscript to ensure replication. A reproducible tutorial for each experiment is accessible at https://pianno-tutorials.readthedocs.io/en/latest/index.html.
Randomization	The study is exploratory and descriptive to demonstrate the utility of a computational method, and no case control comparisons were performed, so no randomization was considered.
Blinding	No blinding as no case-control comparisons are made.

Reporting for specific materials, systems and methods

We require information from authors about some types of materials, experimental systems and methods used in many studies. Here, indicate whether each material, system or method listed is relevant to your study. If you are not sure if a list item applies to your research, read the appropriate section before selecting a response.

Materials & experimental systems

n/a	Involved in the study
☒	☐ Antibodies
☒	☐ Eukaryotic cell lines
☒	☐ Palaeontology and archaeology
☒	☐ Animals and other organisms
☒	☐ Clinical data
☒	☐ Dual use research of concern
☒	☐ Plants

Methods

n/a	Involved in the study
☒	☐ ChIP-seq
☒	☐ Flow cytometry
☒	☐ MRI-based neuroimaging
